# Evaluation of the Oncogene Function of GOLPH3 and Correlated Regulatory Network in Lung Adenocarcinoma

**DOI:** 10.3389/fonc.2021.669684

**Published:** 2021-08-23

**Authors:** Tong Zhang, Yue Wang, Yangyang Chen, Shuo Jin, Ying Gao, Dan Zhang, Yonghui Wu

**Affiliations:** Department of Occupational Health, School of Public Health, Harbin Medical University, Harbin, China

**Keywords:** lung adenocarcinoma, GOLPH3, miR-142-5p, TUG1, bioinformatics analysis

## Abstract

**Background:**

Golgi phosphoprotein 3 (GOLPH3) is an oncoprotein localized in the Golgi apparatus. Abnormal GOLPH3 expression is potentially related to carcinogenesis. However, the potential biological regulation network of GOLPH3 in lung adenocarcinoma (LUAD) remains to be determined.

**Methods:**

Expression of GOLPH3 was identified in LUAD *via* TIMER, Oncomine, Lung Cancer Explorer (LCE), Human Protein Atlas (HPA), and UALCAN database. Survival analysis was performed using the Kaplan–Meier plotter. GOLPH3 alterations were analyzed through cBioPortal. LinkedOmics was used to perform functional analysis and predict interacted targets. The protein–protein interaction network was constructed by GeneMANIA. In addition, candidate miRNAs and lncRNAs targeting GOLPH3 were generated to construct competing endogenous RNA (ceRNA) network, and survival analysis of ceRNA was performed using LnCeVar. The mRNA or protein expression of TUG1, miR-142-5p, and GOLPH3 in Beas-2B and LUAD cells was verified using qPCR or Western blotting. CCK-8 assay, wound healing assay, and transwell assay were used to detect the ability of cell proliferation, migration, and invasion.

**Results:**

Overexpression of GOLPH3 was identified in LUAD. UALCAN analysis showed that upregulated GOLPH3 was linked to different pathological features of LUAD patients. Importantly, high GOLPH3 expression indicated a negative correlation with the first progression (FP) in LUAD patients. GOLPH3 alterations were also found. Moreover, co-expressed genes with GOLPH3 were analyzed; and they were involved in ribosome and oxidative phosphorylation pathways. Functional network analysis indicated GOLPH3 regulated T-cell receptor signaling pathway and interferon signaling pathway with kinase and transcription factor targets. Notably, TUG1/miR-142-5p/GOLPH3 affected overall survival of LUAD patients. GOLPH3 expression was decreased in the cells with overexpression of miR-142-5p and TUG1 knockdown. GOLPH3 reduction inhibited cell proliferation, migration, and invasion.

**Conclusions:**

Upregulation of GOLPH3 has a positive correlation with clinicopathological subtypes and poor FP in LUAD. GOLPH3 promoted LUAD progression. Moreover, TUG1 may act as ceRNA to regulate GOLPH3 expression by competitive binding miR-142-5p.

## Introduction

According to Global Cancer Statistics in 2020, lung cancer has the second highest incidence (11.4%) and mortality (18.0%) among 36 malignancies of 185 countries (1). Approximately 40% patients are clinically diagnosed with lung adenocarcinoma (LUAD), which is the most common type of non-small cell lung cancer (NSCLC), accounting for about 85% of all lung cancer cases. The early-onset symptoms of LUAD are not typical, and LUAD has a strong tendency to recur with high risk of invasion and metastasis (2). The currently available treatment for LUAD included surgery, chemotherapy, radiation, and targeted therapy and immunotherapy, of which remarkable success has been achieved according to recent advancements (3). Despite the progress, treatment efficacy remains unsatisfactory (4, 5), with only 4%–17% crude 5-year survival rate (6). The pathogenesis of lung cancer is extremely complex, involving the evolution of cancer cell genomics and molecular characteristics and their interaction with the tumor microenvironment (7). Therefore, it is necessary to actively identify new drug targets by exploring changed gene regulatory networks that play an important role in LUAD.

Golgi phosphoprotein 3 (GOLPH3) is a highly conserved phosphorylated protein, positioning on the Golgi apparatus and vesicle through combining with phosphatidylinositol 4-phosphate (PI4P). It is related to Golgi vesicle-mediated transport, protein glycosylation, and protein sorting. The GOLPH3 gene is situated on human chromosome band 5p13, which is frequently amplified in lung, prostate, and breast cancers and some other human solid tumors (8). Studies have reported that GOLPH3 has as an oncogene role in a variety of tumors involving molecular pathways such as mTOR/70S6K and Wnt/β-catenin (9). Moreover, GOLPH3 overexpression promoted tumor cell proliferation and migration and affected tumor development (10–12). In addition, GOLPH3 was relevant to poor prognosis and chemotherapy resistance in patients (13, 14). Clinical studies have shown that GOLPH3 was a risk factor for NSCLC; however, its specific functional mechanism in LUAD was still unclear.

In recent years, several studies have indicated that non-coding RNAs (ncRNAs), such as miRNAs and lncRNAs, played a vital role in cancer initiation and progression (15). In 2011, Salmena et al. (16) first proposed competing endogenous RNA (ceRNA) hypothesis regarding the regulatory relationship between ncRNA and mRNA. The ncRNA containing miRNA response element (MRE) binds to miRNA to relieve the inhibitory effect of miRNA on target genes. This hypothesis complements the traditional cognition of gene expression regulation. At the RNA level, the role of multiple ncRNAs is added to the classic miRNA regulation of mRNA translation, and it is extended to the ceRNA–miRNA–mRNA network control mode. Increasing investigations regarding ceRNA in human cancers have been carried out, including lung cancer (17). But a recent integrated and comprehensive analysis of ceRNA and mRNAs in lung cancer was still not enough. Therefore, we decided to investigate the specific ceRNA network in LUAD by way of “GOLPH3–miRNA–lncRNA” order pattern.

In this study, we performed a series of bioinformatics analysis of GOLPH3 expression and regulatory networks to further explore its role in the progress of LUAD. The expression and prognostic value of GOLPH3 in LUAD were evaluated. Then genomic alterations of GOLPH3 were analyzed. Meanwhile, biological functional networks were further predicted. Thus, our results may potentially supply a new target for LUAD diagnosis and treatment.

## Materials and Methods

### Differential Expression of GOLPH3 in Lung Adenocarcinoma

To analyze the difference of GOLPH3 mRNA expression in tumors and normal tissues, TIMER (18) and Oncomine (19) were utilized. We selected *p*-value = 0.01 and data type = mRNA as threshold in Oncomine. Then a meta-analysis of GOLPH3 mRNA expression in LUAD and normal lung tissue was performed by Lung Cancer Explorer (LCE) database (20). The forest plot was provided to summarize tumor-normal standardized mean difference. The GOLPH3 protein level in LUAD was detected in the Human Protein Atlas (HPA) database by proteome methods (21). Furthermore, we applied UALCAN (22) to analyze GOLPH3 expression in LUAD based on sample types, gender, cancer stages, nodal metastasis status, and smoking habits.

### Survival Analysis

The Kaplan–Meier plotter (23) is capable of analyzing the prognostic value of genes on survival in tumor types based on Gene Expression Omnibus (GEO) and The Cancer Genome Atlas (TCGA) data. We assessed the effect of GOLPH3 expression on the overall survival (OS), relapse-free survival (RFS), first progression (FP), and post-progression survival (PPS) of LUAD patients using the Kaplan–Meier plotter, based on RNA-seq data of LUAD and GSE31210 data. The hazard ratio (HR) with 95% confidence intervals and log-rank *p*-value were computed.

### Genomic Alteration of GOLPH3 by cBioPortal

We also applied for cBioPortal (http://www.cbioportal.org) (24) to study the GOLPH3 mutation in LUAD. Genomic alteration types, alteration frequency, and protein change in amino acid were analyzed.

### Functional Enrichment Analysis and Targets of GOLPH3

Genes co-expressed with GOLPH3 in LUAD were analyzed statistically using Pearson’s correlation coefficient by LinkedOmics (http://www.linkedomics.org/login.php) (25). LinkedFinder module was utilized to conduct the correlation analysis of GOLPH3 and differently expressed genes. Statistical results were presented in the volcano plots and heat maps. The correlation among the top 3 differently expressed genes was verified through Gene Expression Profiling Interactive Analysis (GEPIA) (http://gepia.cancer-pku.cn/) (26). LinkInterpreter module was used to perform functional analysis of GOLPH3 in LUAD and also predict its interacted target networks of kinases and transcription factors by Gene Set Enrichment Analysis (GSEA). The rank criterion was false discovery rate (FDR) < 0.05, and 1,000 simulations were carried out. The protein–protein interaction network was constructed by GeneMANIA (http://genemania.org/) (27) for the kinases and transcription factors.

### Analysis of LnRNAs and MiRNAs Related to GOLPH3 Expression in Lung Adenocarcinoma

The potentially effect of miRNAs on GOLPH3 in LUAD was identified using LinkedOmics, TargetScan, miRWalk, miRDB, and starBase databases. Then prognostic value analysis of overlapping miRNAs in LUAD was performed using the Kaplan–Meier plotter. The overlapping miRNAs related to OS of LUAD patients were identified as candidate miRNAs. The relationship between candidate miRNA expression and clinicopathological features of LUAD patients was analyzed using the LinkedOmics database. Then lncRNAs related to GOLPH3 in LUAD were identified by Co-LncRNA (28); and lncRNAs related to candidate miRNAs were identified by starBase. The intersection of the above lncRNAs was evaluated. *p* < 0.01 and FDR < 0.01 were considered. The lncRNAs with significant difference in the expression and prognostic value were identified as candidate lncRNAs. Finally, the effect of lncRNA–miRNA–GOLPH3 on survival in LUAD was analyzed by LnCeVar (29).

### Cell Culture and Transfection

A549 cells and Beas-2B cells were obtained from the Cell Bank of the Chinese Academy of Sciences. H1229 and SPC-A1 cells were provided by Harbin Medical University Cancer Hospital. All cells were maintained in Dulbecco’s modified Eagle medium (DMEM). The culture medium contained 10% fetal bovine serum (FBS) and 1% penicillin/streptomycin. Cell transfection was performed using Lipofectamine 2000 (Invitrogen, Carlsbad, CA, USA) as manufacturer’s protocols. Vectors used in the transfection were synthesized by GenePharma (Shanghai, China) and RIBOBIO (Guangzhou, China), including miR-142-5p mimic, mimic negative control (mimic NC), siRNA of TUG1, GOLPH3, and siRNA negative control (si-NC).

### Cell Proliferation Assessment

The transfected A549 cells were seeded into 96-well plates and placed in a humidified incubator with 5% CO_2_ at 37°C. After incubation for 24, 48, or 72 h, 10 µl of CCK-8 (APExBIO, Houston, USA) solution were added into each well and incubated for 1.5 h at 37°C. The absorbance was determined at 490 nm with a microplate reader (VICTOR Nivo; PerkinElmer, Finland).

### Wound-Healing Assay of Cell Migration

The treated cells were inoculated into 6-well plates. When the cells were adherent to approximately 80%, vertical scribing was conducted at the bottom of the plates with 200-µl spiking tips. Then suspended cells were washed three times with phosphate-buffered saline (PBS) and replaced with fresh medium. The images were captured immediately and after 0, 12 or 24 h of incubation with a microscope (CX41-FS, Olympus, Japan). Finally, we observed and calculated the cell migration distance.

### Transwell Assay of Cell Invasion

Briefly, transfected cells were counted and adjusted to the concentration of 1 × 10^5^/ml. Two hundred microliters of cell suspension was added to the upper transwell chamber containing Matrigel-coated membrane (BD Biosciences, USA). Next, we added 500 µl of DMEM (containing 10% FBS) into the lower chamber for 24-h incubation. After culture termination, the floating cells in the upper chamber were wiped with cotton swabs. The cells that had invaded to the lower surface were fixed with 4% paraformaldehyde for 15 min and stained with crystal violet for 20 min. The images were captured, and the number of invaded cells was counted.

### Quantitative Real-Time Polymerase Chain Reaction

Total RNA was extracted from cells by FastPure Cell/Tissue Total RNA Isolation Kit and MiPure Cell/Tissue miRNA Kit (Vazyme, Nanjing, China), and then it was reverse-transcribed to cDNA using the HiScript II Q RT SuperMix for qPCR Kit and miRNA 1st Strand cDNA Synthesis Kit (by stem-loop) (Vazyme, Nanjing, China). The cDNA was used as a template for real-time qPCR; and qRT-PCR was performed on a QuantStudio3 Real-Time PCR System (Applied Biosystems, USA) with ChamQ™ Universal SYBR^®^ qPCR Master Mix (Vazyme, Nanjing, China). β-Actin and U6 were used as loading controls for GOLPH3, TUG1, and miR-142-5p. The relative fold change in expression of the target normalized to expression of the corresponding control was calculated by the comparative Ct method. The primers used in this study are shown as follows: GOLPH3 (forward: 5′-GGGCGACTCCAAGGAAAC-3′ and reverse: 5′-CAGCCACGTAATCCAGATGAT-3′), miR-142-5p (forward: 5′-GCGCGCATAAAGTAGAAAGC-3′ and reverse: 5′-AGTGCAGGGTCCGAGGTATT-3′), TUG1 (forward: 5′-TAGCAGTTCCCCAATCCTTG-3′ and reverse: 5′-CACAAAT TCCCATCATTCC-3′), β-actin (forward: 5′-GCCGACAGG ATGCAGAAGG-3′ and reverse: 5′-TGGAAGGTGGACAGCG AGG-3′), U6 (forward: 5′-GCTTCGGCAGCACATATACTAA AAT-3′ and reverse: 5′-CGCTTCACGAATTTGCGTGTCAT-3′), and GOLPH3-3′UTR (forward: 5′-GCGCCCGCGATCGCG AATTCCTCTGCTCGGGGTGAAC-3′ and reverse: 5′-ACTAGTCTCGAGTTCAGGCACTTTCAAGATCATTG-3′).

### Dual-Luciferase Reporter Assay

The wild-type (Wt) or mutant type (Mut) 3′-UTR of GOLPH3 was cloned into the pEZX-FR02 vectors (ZX002, GeneCopoeia, Guangzhou, China). In the next step, miR-142-5p mimics or miR-NC and the synthesized plasmids were co-transfected into A549 cells using Lipofectamine 2000. After 48 h of transfection, the luciferase activities were examined using the Luc-Pair™ Duo-Luciferase Assay Kit 2.0 (GeneCopoeia, Guangzhou, China).

### Western Blotting

Cells were lysed using radioimmunoprecipitation assay (RIPA) lysis buffer, and then proteins were quantified using bicinchoninic acid (BCA) method. The extracts including 20 μg of proteins were electrophoresed with 10% sodium dodecyl sulfate–polyacrylamide gel electrophoresis (SDS-PAGE) gels and shifted onto polyvinylidene difluoride (PVDF) membranes. The membranes were blocked using 5% non-fat dry milk for 2 h. Subsequently, they were incubated with specific primary antibodies GOLPH3 (1:1,000, ab5790; Abcam) and β-actin (1:2,000, TA811000; OriGene) at 4°C overnight and followed by interaction with secondary antibody (1:10,000, AS014; ABclonal). Finally, target proteins were examined using an enhanced chemiluminescence (ECL) method.

### Statistical Analysis

Data were analyzed by GraphPad Prism 8.0 software and expressed as mean ± SD. Statistical comparisons between groups were performed using the least significant difference t-test or one-way ANOVA. The differences were considered significant at *p* ≤ 0.05.

## Results

### Elevated Expression of GOLPH3 in Lung Adenocarcinoma

**Figure 1:**
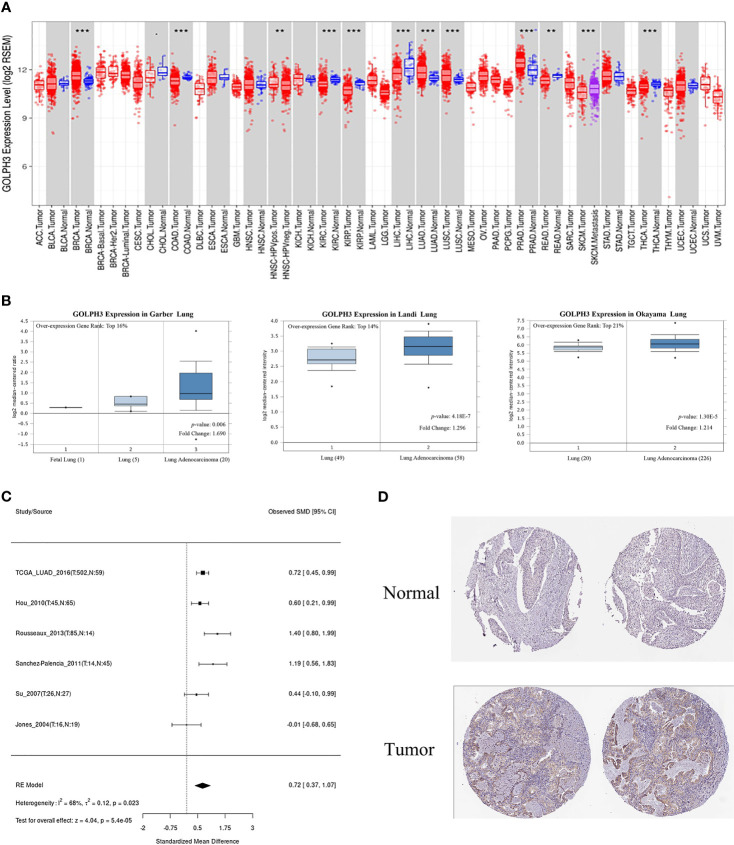
The expression of GOLPH3 in LUAD. **(A)** GOLPH3 mRNA expression in human tumors (TIMER). **(B)** GOLPH3 mRNA expression in Garber lung, Landi lung, and Okayama lung (Oncomine). **(C)** The meta-analysis result of GOLPH3 mRNA expression in LUAD (LCE). **(D)** The protein expression of GOLPH3 in LUAD tissues (HPA). LUAD, lung adenocarcinoma; LCE, Lung Cancer Explorer; HPA, Human Protein Atlas. **p < 0.01, ***p < 0.001.

**Figure 2:**
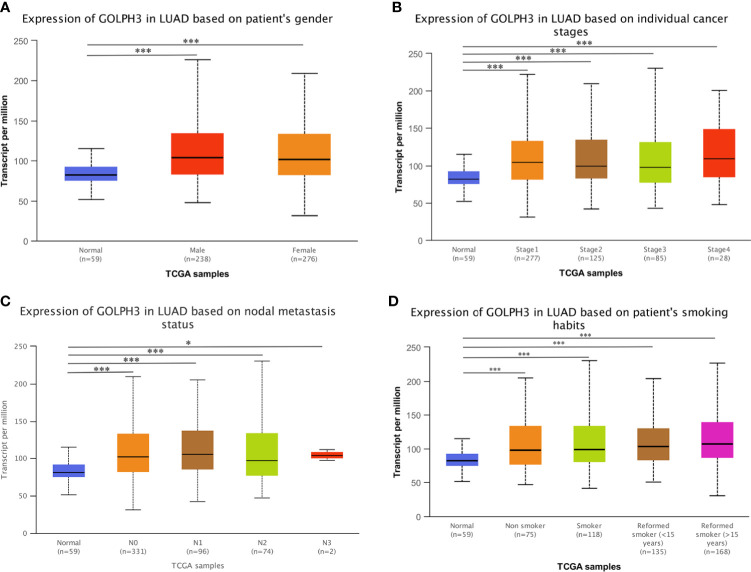
GOLPH3 transcription in subgroups of LUAD patients (UALCAN). **(A)** The relative expression of GOLPH3 in normal individuals of either gender and in male or female lung adenocarcinoma patients. **(B)** The relative expression of GOLPH3 in normal individuals or in LUAD patients in stage 1, 2, 3, or 4. **(C)** The relative expression of GOLPH3 in normal or in LUAD patients in nodal metastasis status N0, N1, N2, or N3. **(D)** The relative expression of GOLPH3 in normal or in non-smoker, smoker, reformed smoker (<15 years), or reformed smoker (>15 years) LUAD patients. The t-test was used to estimate the significance of difference in gene expression levels between groups. Data are mean ± SE. *p < 0.05, ***p < 0.001. LUAD, lung adenocarcinoma. All results are available online.

First, we evaluated GOLPH3 expression in tumors and adjacent normal tissues through the TIMER database to confirm whether GOLPH3 mRNA expression level correlated with human cancers. Comparison results are presented in **Figure 1A**. GOLPH3 mRNA expression was significantly higher in multiple cancerous tissues (including LUAD) compared with normal tissues. And results of Oncomine and LCE databases showed higher GOLPH3 expression in LUAD than normal lung tissues (**Figures 1B, C**). Then we validated the protein expression of GOLPH3 by immunohistochemical (IHC) staining data from HPA database. Results also showed that LUAD tissues have higher GOLPH3 expression than normal tissues (**Figure 1D**). Next, subgroup analysis based on different pathological features of 574 LUAD samples presented evaluated expression of GOLPH3. The transcription level of GOLPH3 had a significant increase in LUAD patients based on gender, cancer stages, nodal metastasis status, and smoking habits (**Figure 2**). These data suggested that high GOLPH3 expression had wide clinical significance in LUAD.

### GOLPH3 Expression Was Associated With Survival in Lung Adenocarcinoma

**Figure 3:**
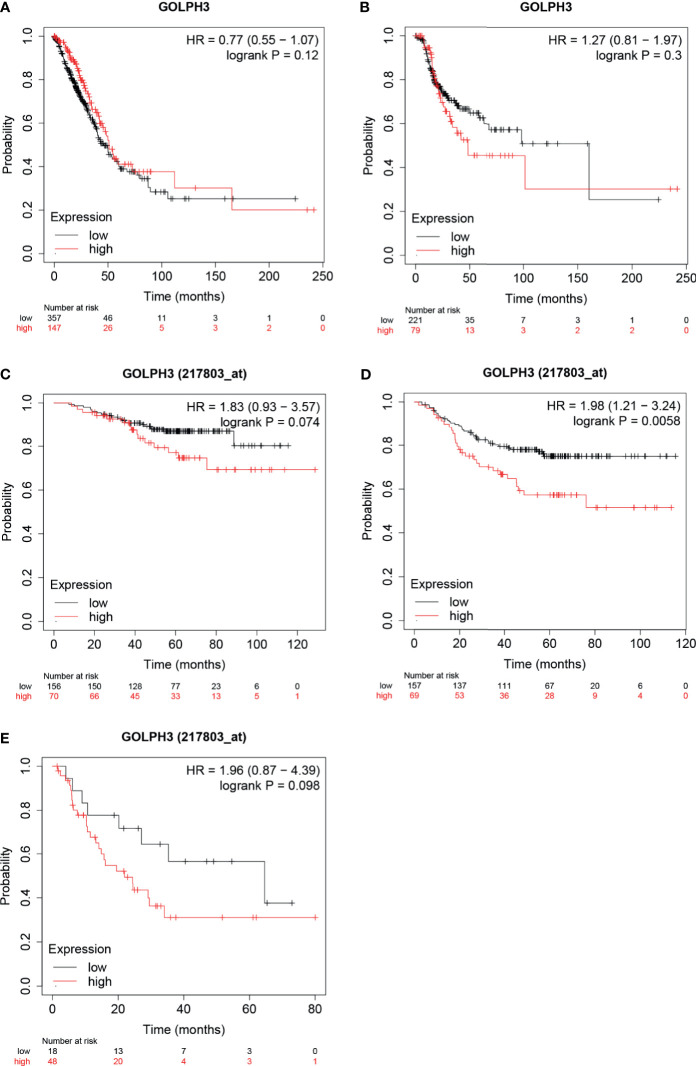
Survival analysis of GOLPH3 (Kaplan–Meier plotter). **(A)** Overall survival (OS) in LUAD. **(B)** Relapse-free survival (RFS) in LUAD. **(C–E)** OS, first progression (FP), and post-progression survival (PPS) in GSE31210 cohort, respectively. LUAD, lung adenocarcinoma. All results are available online.

To evaluate the prognostic value of GOLPH3, the Kaplan–Meier plotter was applied. According to low and high expression of GOLPH3, the OS, RFS, FP, and PPS for LUAD patients were obtained. Results indicated that high GOLPH3 expression was correlated with relatively good prognosis for OS, but poor prognosis for RFS, but the difference was not significant (**Figures 3A, B**). However, further analyses of GSE31210 showed that low GOLPH3 expression group had significantly better OS, FP, and PPS than the high expression group, but only FP showed the significant difference (log-rank *p* = 0.0058) (**Figures 3C–E**). The above data suggested that GOLPH3 expression level possibly may be a factor affecting the survival of LUAD.

### Genomic Mutation of GOLPH3 in Lung Adenocarcinoma

**Figure 4:**
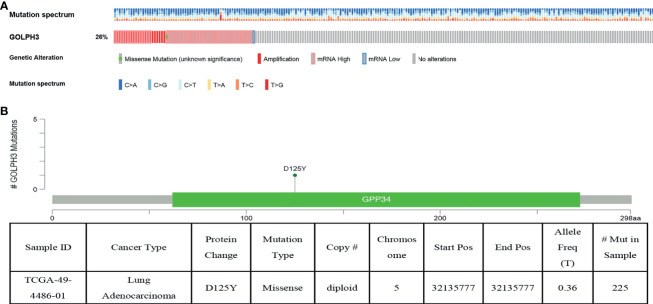
GOLPH3 genomic alterations in LUAD (cBioPortal). **(A)** Oncoprint of GOLPH3 alterations. **(B)** The missense mutation of GOLPH3 amino acids. LUAD, lung adenocarcinoma. All results are available online.

We then determined the GOLPH3 alterations in LUAD patients using the cBioPortal database. GOLPH3 was altered in 60 of 230 (26%) LUAD patients (**Figure 4A**). These alterations were amplification in six cases (2.61%), mRNA upregulation in 36 cases (15.65%), and multiple alterations in 17 cases (7.39%) (**Table 1**). In addition, missense mutation of GOLPH3 resulted in the amino acid change, which was aspartic acid (D) 125 replaced by tyrosine (Y) (**Figure 4B**). These results indicated that genetic alteration of GOLPH3 could be found in LUAD, which might play a significant role in tumorigenesis of LUAD.

**Table 1:** Summary for lung adenocarcinoma gene altered in 26.09% of 230 cases.

Alteration	Frequency
Amplification	2.61% (6 cases)
mRNA high	15.65% (36 cases)
mRNA low	0.43% (1 case)
Multiple alterations	7.39% (17 cases)

### Co-Expression Networks of GOLPH3 in Lung Adenocarcinoma

**Figure 5:**
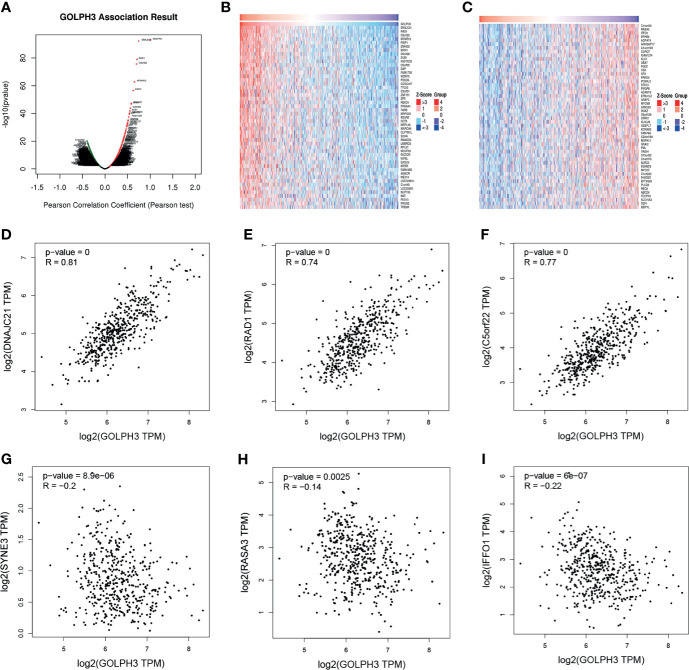
GOLPH3 co-expression genes in LUAD (LinkedOmics and GEPIA). **(A)** The global genes highly correlated with GOLPH3 in LUAD were identified using Pearson’s test. **(B, C)** Heat maps show genes positively and negatively relevant to GOLPH3 (top 50). Red indicates positively correlated genes, and blue indicates negatively correlated genes. **(D–F)** Top 3 genes positively correlated with GOLPH3. **(G–I)** Top 3 genes negatively correlated with GOLPH3. LUAD, lung adenocarcinoma; GEPIA, Gene Expression Profiling Interactive Analysis. All results are available online.

To further understand the biological role of GOLPH3 in LUAD, we used the function module of LinkedOmics to examine GOLPH3 co-expression genes in LUAD cohort. The volcano plot showed 1,731 positively correlated genes and 3,585 negatively correlated genes (FDR < 0.01) (**Figure 5A**). The heat map revealed the 50 significant genes positively and negatively correlated with GOLPH3 (**Figures 5B, C**). GOLPH3 expression was significantly positively relevant to DNAJC21, RAD1, and C5orf22 (**Figures 5D–F**) and negatively relevant to C14orf49, RASA3, and IFFO1 (**Figures 5G–I**).

**Figure 6:**
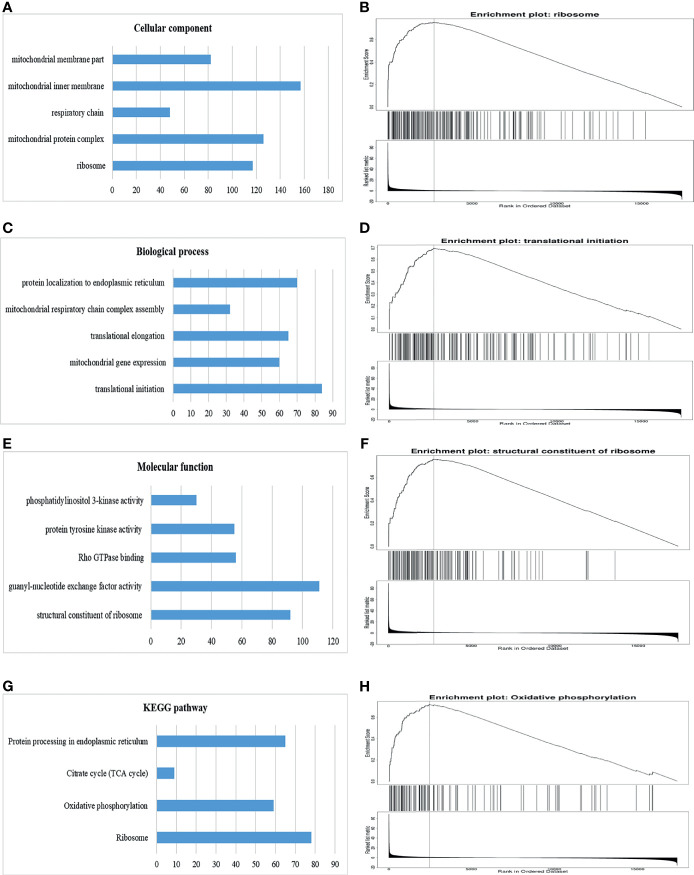
Significantly enriched GO annotations and KEGG pathways of GOLPH3 using GSEA in LUAD (LinkedOmics). **(A)** Cellular component (CC). **(B)** Enrichment plot of ribosome in CC. **(C)** Biological process (BP). **(D)** Enrichment plot of translational initiation in BP. **(E)** Molecular function (MF). **(F)** Enrichment plot of structural constituent of ribosome in MF. **(G)** KEGG pathway. **(H)** Enrichment plot of oxidative phosphorylation pathway in KEGG. GSEA, Gene Set Enrichment Analysis; LUAD, lung adenocarcinoma; GO, Gene Ontology; KEGG, Kyoto Encyclopedia of Genes and Genomes. All results are available online.

Significant Gene Ontology (GO) annotation analysis by GSEA suggested that GOLPH3 co-expression genes were localized mainly in ribosome, mitochondrial protein complex, respiratory chain, mitochondrial inner membrane, and mitochondrial inner membrane part (**Figures 6A, B**), where they participate primarily in translational initiation, mitochondrial gene expression, translational elongation, mitochondrial respiratory chain complex assembly, and protein localization to endoplasmic reticulum (**Figures 6C, D**), suggesting the effects on structural constituent of ribosome, guanyl-nucleotide exchange factor activity, Rho GTPase binding, protein tyrosine kinase activity, and phosphatidyl inositol 3-kinase activity (**Figures 6E, F**). Kyoto Encyclopedia of Genes and Genomes (KEGG) pathway analysis found enrichment in the ribosome and oxidative phosphorylation (**Figures 6G, H**).

### GOLPH3 Networks of Kinase and Transcription Factor Targets in Lung Adenocarcinoma

To investigate targets of GOLPH3 in LUAD, we used GSEA to further analyze kinase and transcription factor target networks of positively associated gene sets. As shown in **Table 2**, the top 5 most significant kinase targets were kinase_LCK (LCK proto-oncogene, Src family tyrosine kinase), kinase_SYK (spleen associated tyrosine kinase), kinase_FYN (FYN proto-oncogene, Src family tyrosine kinase), kinase_PRKD1 (protein kinase D1), and kinase_PRKCA (protein kinase C alpha). The top 5 most significant transcription factor targets included V$IRF_Q6, V$NFKB_Q6_01, V$AML_Q6, STTTCRNTTT_V$IRF_Q6, and V$NFKB_C.

**Table 2:** The kinases and transcription factors—target networks of GOLPH3 in lung adenocarcinoma.

Enriched category	Gene set	LeadingEdgeNum	FDR
Kinase target	Kinase_LCK	27	0.0000000
	Kinase_SYK	23	0.0017495
	Kinase_FYN	32	0.0116631
	Kinase_PRKCA	92	0.0153564
	Kinase_PRKD1	18	0.0177279
Transcription factor	V$IRF_Q6	92	0.00049911
Target	GGGNNTTTCC_V$NFKB_Q6_01	54	0.00066549
	V$AML_Q6	87	0.00099823
	STTTCRNTTT_V$IRF_Q6	74	0.00099823
	V$NFKB_C	112	0.00219610

LeadingEdgeNum, the number of leading edge genes; FDR, false discovery rate from Benjamini and Hochberg from Gene Set Enrichment Analysis (GSEA); V$, the annotation found in Molecular Signatures Database (MSigDB) for transcription factors (TFs).

**Figure 7:**
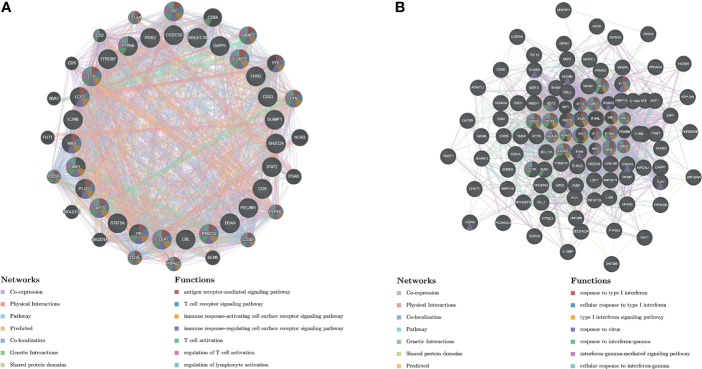
Protein–protein interaction network of LCK kinase and transcription factor IRF-target networks (GeneMANIA). PPI network and functional analysis indicating the gene set that was enriched in the target network of **(A)** LCK kinases and **(B)** transcription factor IRF. Distinct colors of the network edge indicate the bioinformatics methods applied; distinct colors for the network nodes indicate the biological functions of the sets of enrichment genes. PPI, protein–protein interaction.

The protein–protein interaction network constructed by GeneMANIA revealed correlation among genes for the kinase_LCK and V$IRF_Q6. The gene set enriched for kinase_LCK was responsible mainly for regulating T-cell receptor signaling pathway, immune response-activating cell surface receptor signaling pathway, and T-cell activation, while V$IRF_Q6 was responsible mainly for type I interferon (IFN) signaling pathway and IFN-gamma-mediated signaling pathway (**Figure 7**).

### MiR-142-5p Could Downregulate GOLPH3 Expression Following Good Prognosis in Lung Adenocarcinoma

**Figure 8:**
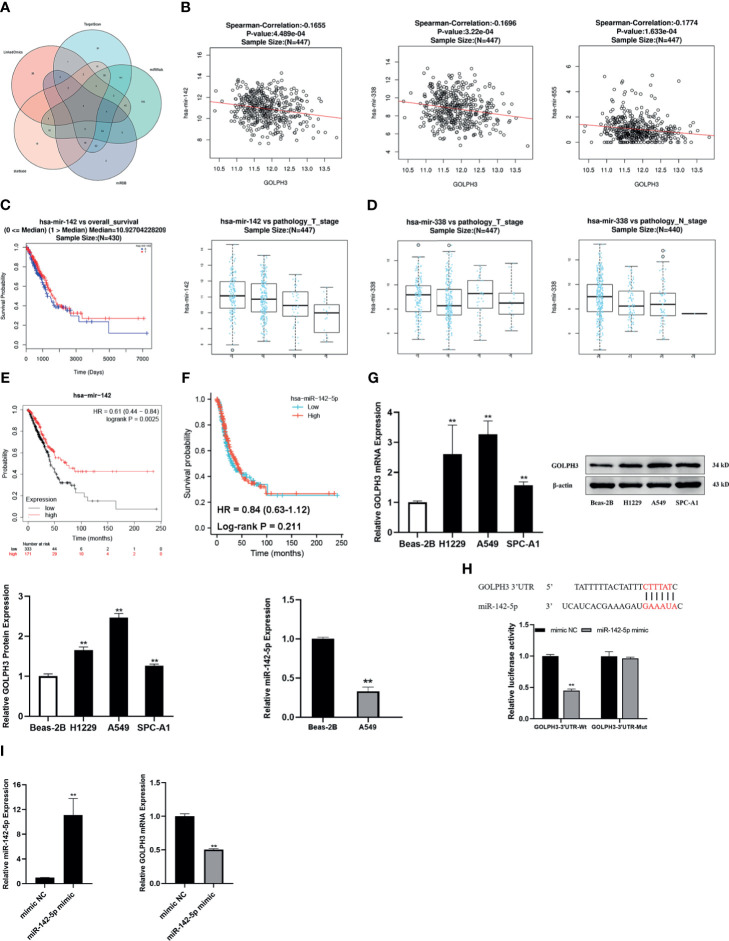
Construction of miRNA–GOLPH3 network. **(A)** Candidate miRNAs (miR-142-5p, miR-183-5p, miR-338-5p, and miR-655-3p) are predicted by LinkedOmics, TargetScan, miRWalk, miRDB, and starBase. **(B)** The expression relationship between miRNAs and GOLPH3. **(C)** The clinical relevance of miR-142 with LUAD in LinkedOmics. **(D)** The clinical relevance of miR-338 with LUAD in LinkedOmics. **(E)** The overall survival (OS) of miR-142 in LUAD by Kaplan–Meier plotter. **(F)** The first progression (FP) of miR-142-5p in LUAD using TCGA database. **(G)** The expression of miR-142-5p and GOLPH3 in Beas-2B and LUAD cells using qRT-PCR and Western blotting. **(H)** The luciferase reporter assay of A549 cells was transfected with the GOLPH3-3′-UTR-Wt or GOLPH3-3′-UTR-Mut in the miR-142-5p binding sites. **(I)** The expression of miR-142-5p and GOLPH3 in A549 cells with overexpression of miR-142-5p using qRT-PCR. **p < 0.01. LUAD, lung adenocarcinoma; TCGA, The Cancer Genome Atlas.

We predicted 103 miRNAs correlated with GOLPH3 in 450 samples using the LinkedOmics database of LUAD (FDR < 0.05). Then we investigated miRNAs associated with GOLPH3 from the “TargetScan,” “miRWalk,” “miRDB,” and “starBase” databases. Finally, four overlapping miRNAs (miR-142-5p, miR-183-5p, miR-338-5p, and miR-655-3p) were obtained (**Figure 8A**). The expression and prognosis relationship between miRNAs and GOLPH3 were detected. Our results showed a significant negative correlation between miR-142, miR-338, and miR-655 expression and GOLPH3 (**Figure 8B** and **Table 3**). **Figure 8C** shows that high expression of miR-142 is significantly associated with pathology_T_stage (*p* = 1.921E−04) and OS (*p* = 3.334E−02), and high expression of miR-338 was significantly associated with pathology_T_stage (*p* = 1.684E−02) and pathology_N_stage (*p* = 2.345E−02) (**Figure 8D**), but not OS. Finally, we applied the Kaplan–Meier plotter database to observe the prognostic value of miR-142 again. The similar result was also found in that patients with overexpression of miR-142 had significantly good prognosis (**Figure 8E**). We also utilized TCGA database to analyze the FP of miR-142-5p in LUAD, but the difference was not statistically significant (*p* = 0.211) (**Figure 8F**).

**Table 3:** The miRNAs targeting GOLPH3 in lung adenocarcinoma.

MiRNAs	Correlation based on Spearman	*p*-Value	FDR (BH)
MiR-142-5p	−0.165519032	0.00044893	0.006852531
MiR-338	−0.169595563	0.000322026	0.00531671
MiR-655-3p	−0.177390323	0.000163319	0.003422352
MiR-183-5p	0.224513251	1.73539E−06	0.000116994

FDR (BH), false discovery rate (Benjamini and Hochberg).

### Relationship Between MiR-142-5p and GOLPH3 in Lung Adenocarcinoma Cells

The effect of miR-142-5p on GOLPH3 expression was further verified in LUAD cells. Three LUAD cell lines and a normal human bronchial epithelial cell line were used in this study. As shown in **Figure 8G**, GOLPH3 was significantly upregulated in LUAD cells compared with Beas-2B cells (*p* < 0.01). And then we detected the expression of miR-142-5p in A549 cells in which GOLPH3 expression had the most significant statistical difference compared with Beas-2B cells. MiR-142-5p was remarkably downregulated in A549 cells compared with Beas-2B cells (*p* < 0.05). Our results were consistent with the data obtained from bioinformatics analysis. Luciferase reporter assays revealed that the overexpression of miR-142-5p obviously decreased the luciferase activity of Wt-3′UTR of GOLPH3 but not Mut-3′UTR of GOLPH3 in A549 cells (**Figure 8H**), indicating that miR-142-5p can bind to the GOLPH3 3′UTR. Meanwhile, GOLPH3 expression was suppressed in A549 cells with overexpression of miR-142-5p (**Figure 8I**), which further intimated that miR-142-5p targeted negative regulation of GOLPH3.

### TUG1 May Upregulate GOLPH3 Targeted by Binding to MiR-142-5p

**Figure 9:**
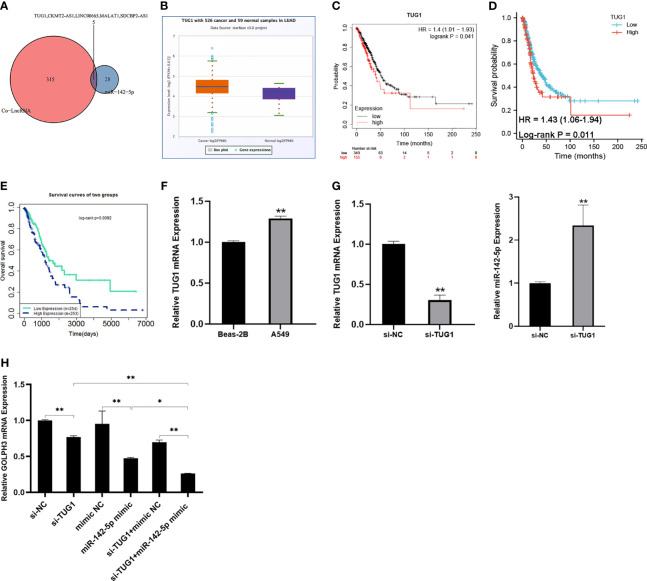
Construction of lncRNA–miRNA–GOLPH3 network. **(A)** Candidate lncRNAs are predicted by co-LncRNA and starBase. **(B)** The expression of TUG1 in LUAD by starBase. **(C)** The overall survival (OS) of TUG1 in LUAD by Kaplan–Meier plotter. **(D)** The first progression (FP) of TUG1 in LUAD using TCGA database. **(E)** The overall survival (OS) of TUG1-miR-142-5p-GOLPH3 in LUAD by LnCeVar. **(F)** The expression of TUG1 in Beas-2B and A549 cells using qRT-PCR. **(G)** The expression of TUG1 and miR-142-5p in A549 cells with knockdown of TUG1 using qRT-PCR. **(H)** The expression of GOLPH3 in A549 cells with siTUG1 and miR-142-5p mimic using qRT-PCR. *p < 0.05, **p < 0.01. LUAD, lung adenocarcinoma; TCGA, The Cancer Genome Atlas.

Then we predicted five candidate lncRNAs related to GOLPH3 and miR-142-5p (**Figure 9A**). Among them, there was a higher expression of TUG1 in LUAD than normal tissues (**Figure 9B**). OS and FP of the patients who had high expression of TUG1 were poor (*p* < 0.05) (**Figures 9C, D**). Besides, the effect of TUG1-miR-142-5p-GOLPH3 on survival in LUAD was analyzed by LnCeVar. It affected OS of patients (**Figure 9E**).

### Relationship Between TUG1 and MiR-142-5p, and GOLPH3 in A549 Cells

Additionally, to determine whether TUG1 regulated GOLPH3 expression in A549 cells by sponging miR-142-5p, the expression of GOLPH3 and miR-142-5p in the TUG1-inhibited A549 cells was examined by qRT-PCR. Our results revealed a significant increase TUG1 in A549 cells compared with Beas-2B cells (**Figure 9F**) and an obvious reduction GOLPH3 in TUG1-deleted A549 cells, while the opposite result of miR-142-5p expression was observed (**Figures 9G, H**). Importantly, when silencing TUG1 and overexpressing miR-142 at the same time, the expression of GOLPH3 was significantly reduced compared with the group of silencing TUG1 or overexpressing miR-142 alone (**Figure 9H**).

### GOLPH3 Silencing Repressed A549 Cell Proliferation, Migration, and Invasion

**Figure 10:**
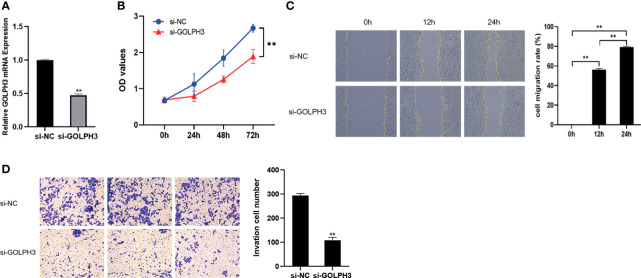
GOLPH3 silencing suppressed A549 cell proliferation, migration, and invasion. **(A)** Relative GOLPH3 expression by qRT-PCR in cells transfected with si-GOLPH3. **(B)** CCK-8 assay was used to detect cell proliferation. **(C)** Wound-healing assay was used to detect cell migration. **(D)** Transwell assay was used to detect cell invasion. **p < 0.01.

To further confirm the effect of GOLPH3 on LUAD progression, we downregulated GOLPH3 expression using siRNA by transfection. Si-GOLPH3 significantly inhibited the expression of GOLPH3 in A549 cells (**Figure 10A**). Moreover, we observed that GOLPH3 reduction markedly suppressed cell proliferation (**Figure 10B**). Meanwhile, the migration (**Figure 10C**) and invasion (**Figure 10D**) abilities were decreased during GOLPH3 silencing in A549 cells.

## Discussion

As defined first-in-class Golgi oncoprotein, GOLPH3 activation accelerated malignant progression of LUAD (30). In order to further assess the structure and possible function of GOLPH3 in LUAD, we carried out a comprehensive bioinformatics analysis using TCGA and GEO datasets. In this study, based on RNA-seq and IHC data, we found that the mRNA and protein expression levels of GOLPH3 were much higher in LUAD than normal tissues. Besides, high GOLPH3 expression was closely related to the clinical significance and poor FP in LUAD. These results suggest GOLPH3 plays a significant role in the development of LUAD.

Genome copy number change is one of the important causes of tumorigenesis. Copy number variations have significant genomic significance and commonly involved in lung cancer (31). It has been reported that GOLPH3 was located on chromosome band 5p13 in NSCLC, and the depletion of GOLPH3 reverts cell oncogene transformation (8). In this study, GOLPH3 gene alteration was also found in LUAD, such as copy number amplification and missense mutation, which is consistent with previous studies.

In the following analysis, we took advantage of GSEA to explore potential function of GOLPH3 in LUAD. We found that GOLPH3 participated primarily in translational and biological regulations and is involved in oxidative phosphorylation signaling pathways. The Golgi apparatus is an important part in cellular material metabolism and protein secretion pathways. GOLPH3, as a new peripheral membrane protein located in the *trans*-Golgi network, was closely related to the structure and function of Golgi (32). GOLPH3 had an influence on vesicle transport level and cell secretion function in Golgi (33). It was reported that high GOLPH3 expression promoted tumor growth and metastasis-related protein secretion (34). Furthermore, GOLPH3 was rich in mitochondrial, playing an important role in tumor metabolism (35, 36), including GOLPH3-mediated autophagy resistance and mitochondrial biogenesis in breast cancer (37). Our findings exactly indicated that GOLPH3 was mainly correlated with mitochondrial function in LUAD.

For further mining the potential regulators for GOLPH3, we found that GOLPH3 was in connection with kinases LCK, SYK, FYN, PRKD1, and PRKCA in LUAD. LCK and FYN were non-receptor tyrosine kinases of Src family kinases (SFKs), which are associated with diverse receptors to mediate cell growth and adhesion (38). Consequently, SFKs are perceived as cancer therapy targets owing to over-hyper-activation in malignancies following poor patient prognosis. Indeed, LCK overexpression was also found in NSCLC cells (39). In LUAD, GOLPH3 possibly regulates T-cell receptor signaling pathway, immune response-activating cell surface receptor signaling pathway, and T-cell activation *via* LCK kinase.

Next, we identified IRF, NFKB, and AML as the main transcription factors responsible for GOLPH3. IFN gene expression is regulated by IFN regulatory factors (IRFs) and affects the bioactivities such as cell growth and differentiation, regulating immune function by activating related signal transduction pathway after being mediated through IRF and IFN-stimulated gene (ISG) products. IRF was often found to be epigenetically modified during carcinogenesis (40), such as high methylation in lung cancer (41). Consistent with the above results, we predicted that GOLPH3 may regulate type I IFN signaling pathway and IFN-gamma-mediated signaling pathway through IRF in LUAD. Further investigations are needed to test the hypothesis.

To clearly explore the role of GOLPH3 in the pathogenesis of LUAD, we also focused on the mechanism of post-transcriptional regulation of GOLPH3. MiRNA, as a small endogenous ncRNA molecule, can negatively regulate gene expression by binding to complementary sequences in the 3′ untranslated region (UTR) of their target mRNAs, resulting in degradation of the mRNA transcript or suppression of the protein translation process (42). A strong relationship between miRNAs and GOLPH3 in human cancers has been reported in the literature. For instance, Zhang et al. suggested that miR34a/GOLPH3 Axis abrogated urothelial bladder cancer chemoresistance (43). Liu et al. found that miR-134 suppresses cell proliferation in gastric cancer cells *via* targeting of GOLPH3 (44). Moreover, miRNA also can act a biological role in the nucleus by binding to the promoter region of the target gene, resulting in silencing or overexpression of the target gene (45). Experiments have confirmed that miR-373 can bind to the promoter of targeted mRNA and activate gene expression (46). In this study, results showed a negative correlation between miR-142-5p and GOLPH3 expression in LUAD. Survival analysis revealed that patients with higher expression of miR-142-5p had better prognosis in LUAD. A previous study had reported the tumor-suppressive roles of miR-142-5p in LUAD. MiR-142-5p was significantly downregulated in LUAD patients and may act as a new non-invasive biomarker for the early diagnosis of LUAD (47). Downregulation of miR-142-5p leads to upregulation of HOXD8 and caused drug resistance in lung cancer cells (48). Thus, miR-142-5p may function as tumor suppressors in LUAD.

In recent decades, a growing number of lncRNAs have been found to be key regulators in tumor initiation and progression. LncRNA has a complex secondary and tertiary structure. It can regulate gene expression by combining with DNA, RNA, and protein (49, 50). LncRNA has four main functions (51): signal (functioning as indicators of transcriptional activity) (52), guide (recruiting chromatin modifying enzymes to target genes to direct their *cis* or *trans* chromatin remodelling and epigenetic regulation), decoy (binding of lncRNAs as “miRNA sponges” to miRNAs and recruitment of transcription factors or protein factors away from chromatin to the nucleus), and scaffold (mediating the stability of ribonucleoprotein complexes). There are already reports about regulation of GOLPH3 by lncRNA. For example, Shan et al. demonstrated that LINC00657 targeted miR-590-3p/GOLPH3 axis to affect breast cancer cell apoptosis (53). Hence, we further predicted five lncRNAs related to GOLPH3 and miR-142-5p. By combining expression analysis and survival analysis for these lncRNAs in LUAD using TCGA data, only TUG1 was defined as the key lncRNA. TUG1 is a recently identified oncogenic lncRNA, the aberrant upregulation of which has been detected in different types of cancer (54). TUG1 was significantly overexpressed in CRC patients (55), functioned as an oncogene in osteosarcoma by competitive binding with miR-335-5p (56). Indeed, knockdown of TUG1 suppressed LUAD cell viability and promoted cell apoptosis (57). Meanwhile, we found that TUG1-miR-142-5p-GOLPH3 network has a prognostic impact on LUAD patients. It suggested that TUG1 may participate in the development of LUAD through miR-142-5p/GOLPH3 axis.

The expression of TUG1, miR-142-5p, and GOLPH3 in A549 cells was also examined. The mRNA expression of GOLPH3 in A549 cells was higher than that in the normal Beas-2B cells, and GOLPH3 silencing hampered cell proliferation, invasion, and migration, suggesting that GOLPH3 might serve as an oncogene of LUAD. The same is true for TUG1 expression. By contrast, downregulated expression of miR-142-5p was observed in A549 cells compared with the normal cells, which indicated that miR-142-5p might function as a suppressive miRNA in LUAD. In addition, markedly decreased GOLPH3 expression was observed in A549 cells with overexpression of miR-142-5p and knockdown of TUG1, compared with overexpression of miR-142-5p or silencing TUG1 alone. All these expression data were consistent with the bioinformatics results.

Taken together, the data of our study clarify the relationship of GOLPH3 and LUAD from multiple perspectives. The novel insights obtained from bioinformatics analysis are helpful to further experiments *in vivo* and *in vitro* and clinical studies to prove the specific value and meaning of GOLPH3 in LUAD.

## Data Availability Statement

The original contributions presented in the study are included in the article/supplementary material. Further inquiries can be directed to the corresponding author.

## Author Contributions

TZ, YW, and YHW conceived the idea of the study. TZ conducted the data analysis and drafted and revised the article. YC, SJ, YG, and DZ participated in the experiments and revised the article. All authors contributed to the article and approved the submitted version.

## Funding

This study was supported by the projects from the National Natural Science Foundation of China (81973010).

## Conflict of Interest

The authors declare that the research was conducted in the absence of any commercial or financial relationships that could be construed as a potential conflict of interest.

## Publisher’s Note

All claims expressed in this article are solely those of the authors and do not necessarily represent those of their affiliated organizations, or those of the publisher, the editors and the reviewers. Any product that may be evaluated in this article, or claim that may be made by its manufacturer, is not guaranteed or endorsed by the publisher.
